# Conserving Plants in Gene Banks and Nature: Investigating Complementarity with *Trifolium thompsonii* Morton

**DOI:** 10.1371/journal.pone.0105145

**Published:** 2014-08-14

**Authors:** Stephanie L. Greene, Theodore J. Kisha, Long-Xi Yu, Mauricio Parra-Quijano

**Affiliations:** 1 National Center for Genetic Resource Preservation, United States Department of Agriculture-Agricultural Research Service, Fort Collins, Colorado, United States of America; 2 Western Regional Plant Introduction Station, United States Department of Agriculture-Agricultural Research Service, Pullman, Washington, United States of America; 3 Departamento de Biologıa Vegetal, Universidad Politecnica de Madrid, Madrid, Spain; USDA- ARS, United States of America

## Abstract

A standard conservation strategy for plant genetic resources integrates *in situ* (on-farm or wild) and *ex situ* (gene or field bank) approaches. Gene bank managers collect *ex situ* accessions that represent a comprehensive snap shot of the genetic diversity of *in situ* populations at a given time and place. Although simple in theory, achieving complementary *in situ* and *ex situ* holdings is challenging. Using *Trifolium thompsonii* as a model insect-pollinated herbaceous perennial species, we used AFLP markers to compare genetic diversity and structure of *ex situ* accessions collected at two time periods (1995, 2004) from four locations, with their corresponding *in situ* populations sampled in 2009. Our goal was to assess the complementarity of the two approaches. We examined how gene flow, selection and genetic drift contributed to population change. Across locations, we found no difference in diversity between *ex situ* and *in situ* samples. One population showed a decline in genetic diversity over the 15 years studied. Population genetic differentiation among the four locations was significant, but weak. Association tests suggested infrequent, long distance gene flow. Selection and drift occurred, but differences due to spatial effects were three times as strong as differences attributed to temporal effects, and suggested recollection efforts could occur at intervals greater than fifteen years. An effective collecting strategy for insect pollinated herbaceous perennial species was to sample >150 plants, equalize maternal contribution, and sample along random transects with sufficient space between plants to minimize intrafamilial sampling. Quantifying genetic change between *ex situ* and *in situ* accessions allows genetic resource managers to validate *ex situ* collecting and maintenance protocols, develop appropriate recollection intervals, and provide an early detection mechanism for identifying problematic conditions that can be addressed to prevent further decline in vulnerable *in situ* populations.

## Introduction

Since utilization of plant genetic resource (PGR) conservation is an important aim, it’s long been recognized that effective strategies need to integrate *in situ* (on-farm or in the wild) and *ex situ* (gene or field bank) approaches. *In situ* conservation allows the natural trajectory of evolution to continue to mold genetic variation, and ensures sustained access to genetically adapted populations. An important strength of *ex situ* conservation is that it readily provides the diverse germplasm needed by plant breeders, natural research managers and basic and applied researchers. In contrast to *in situ* populations, *ex situ* accessions provide a genetic snapshot, reflecting a wild (or on-farm) population’s adaptation to the biotic and abiotic conditions it was collected in. The static nature of *ex situ* conservation is frequently cast as a disadvantage; however, there is evidence that these snapshots conserve alleles that can be lost from *in situ* populations. For example, in a comparison of barley (*Hordeum vulgare* L.) landraces conserved *in situ* (on farm) in Morocco, and *ex situ* (since 1985), for resistance to current strains of powdery mildew (*Blumeria graminis* f.sp. *hordei*), *ex situ* accessions had more qualitative resistance than *in situ* accessions, for some pathotypes of powdery mildew, due to rare resistance genes being preserved *ex situ*, but lost *in situ* [1]. Other studies have reported the conservation of molecular marker bands in *ex situ* samples that have disappeared from current *in situ* populations [2], [3]. These studies reaffirm the general consensus that the most effective PGR conservation strategies need to integrate both an *in situ* and *ex situ* approach [4]–[6].

Gene bank curators and managers generally aim to have *ex situ* samples that represent the inherent diversity of *in situ* populations at the time they are sampled. The literature suggests this might not be a straight forward proposition. *In situ* and *ex situ* accessions were compared in maize (*Zea mays* L.) landraces using morphological [7] and SSR markers [8]. *Ex situ* accessions of American Indian Hopi landraces differed significantly from their *in situ* counterparts that had been maintained on farm in Arizona and the differences were attributed to original collecting bottlenecks and seed multiplication in a different environment (Iowa) [7]. In contrast, similar levels of genetic diversity and insignificant differentiation between *in situ* and *ex situ* samples of Jala, a Mexican maize race, were attributed to good initial sampling and effective regeneration practices [8]. Bean (*Phaseolus vulgare* L.) landraces conserved *ex situ* and on farm were compared using SSR markers [3] and phenotypic and development traits [9]. Both studies found that gene diversity was significantly less in *ex situ* subpopulations. SSR data indicated significant genetic differentiation in *ex situ* subpopulations as well as loss of alleles, gain of new alleles, and reduction of rare alleles and increase of common alleles [3]. Significant changes in yield, 100 seed weight, maturity and leaf area were also reported [9]. Both studies concluded that changes in genetic and phenotypic makeup in *ex situ* subpopulations could be attributed to regeneration practices. Using ISSR markers similar results were found in Oca (*Oxalis tuberosa* Mol), a tuber crop species grown in the Andes [10].

The challenges of maintaining complementary samples of wild species using *ex situ* and *in situ* methods are also evident. RAPDs were used to examine three remaining natural populations and one *ex situ* (conserved in a botanical garden field bank) subpopulation of *Vatica guangxiensis* (X. L. Mo), a rare endemic of southwestern China. The *ex situ* subpopulation contained 88.31% of the total genetic variation and was thought to adequately represent extant natural variation [11]. In contrast, RAPDs were used to examine *Parashorea chinensis* H. Wang, a rare endemic timber tree of southwest China and adjacent areas of Laos and Vietnam. The *ex situ* subpopulation (conserved in a field bank) was found to contain only 77.1% of the total variation found in samples taken from seven *in situ* sites. Additional sampling was recommended [12]. RAPDs were also used to assess *ex situ* samples (cultivated as ornamentals) of *Berberidopsis corallina* Hook. f., a threatened vine endemic to southern Chile, and found to only represent the northern part of the natural range of the species [13]. All three studies emphasized the importance of adequate sampling to ensure *ex situ* accessions represent the genetic diversity of wild species. In contrast, *in situ* populations of *Agropyron cristatum* (L.) Gaertn., a widely distributed wind pollinated grass species, were compared with gene bank counterparts collected 28–30 years previously, using SSRs. Although there were individual loci differences for number of alleles and genetic diversity, when averaged across all loci there was no significant difference among recent recollections and *ex situ* stored accessions [14]. RAPDs were used to examine recollections of *Solanum jamesii* Torrey (diploid outcrosser) and *Solanum fendleri* A. Gray (tetraploid inbreeder), crop wild relative (CWR) species of potato (*Solanum tuberosum* L.). Subpopulations were originally sampled in 1958, and recollected in 1978 and 1992. Significant genetic differences were found between all *ex situ* and re-collected *in situ* subpopulations of *S. jamesii*, and 12 of 16 comparisons of *S. fendleri*. Relative differences were attributed to mating system, vulnerability of small populations (<100 plants) to genetic change, and difference in original sampling procedures [2].

These studies emphasize two factors that are important to obtain and maintain *ex situ* samples that reflect an accurate snapshot of *in situ* populations. One, comprehensive initial sampling is imperative at both population and taxon level; and two, changing selection regimes–from either the *ex situ* regeneration/field site, or *in situ* site itself, can contribute to the divergence of *in situ* and *ex situ* accessions. Although a large body of literature outlines collecting parameters to ensure adequate sampling of PGR [15]–[17], and regeneration strategies to minimize genetic change [18]–[20], the literature also reflects how challenging it is to obtain *ex situ* samples that represent *in situ* populations. Recollection, rather than regeneration, may be a useful approach. This has been proposed for wild species that are difficult to regenerate *ex situ*, because natural conditions are too costly to replicate, or the material itself is difficult to propagate [20]. An area that has had little investigation is the frequency that recollection should occur. In other words, how frequently should a snapshot be taken? Recollection frequency is usually driven by declining viability of *ex situ* samples. Should *ex situ* managers also consider how quickly the genetic structure of *in situ* PGR might be changing? Seven farmer varieties of rice, conserved *ex situ* and *in situ* (on farm) over a period of time when production practices changed, were compared using allozymes, agronomic, stress resistance and morphological traits. Although yield and genetic diversity remained the same, traits associated with adaptation to new production practices significantly changed between *ex situ* and on farm subpopulations in a period of seventeen years [21]. Considering that climate change is occurring at a faster pace than predicted [22] and that models predict environmental change will not only drive changes in distribution and species extinction [23], but impact genetic diversity [24]. It is timely investigating how quickly *in situ* populations change over time, and how this may impact *ex situ* resampling.

*Trifolium thompsonii* Morton (2n = 2x = 16) is a restricted endemic herbaceous species that grows on the east slopes of the Cascade Mountains, in central Washington State, USA. The species is ranked as globally imperiled [25] and wild clover species, especially native to the US, have been identified as important to conserve in the USDA National Plant Germplasm System collection [26]. The species is a dominant forb in early seral communities that are disturbed by fire or grazing [27]. Fire has been excluded for nearly a century in the Cascade Mountains and species rarity has been attributed to increased shading due to overstory trees and competition with ground-layer species [28]–[30]. In 1988 the Dinkleman Fire swept through much of the range of *T*. *thomsonii*, effectively returning the area consumed to an early seral community. We used this species as an insect-pollinated, strongly out-crossing model to study population genetic change, since it is diploid, occurs in diverse environments that are undergoing successional change, occurs in populations greater than 10,000 plants, and is readily accessed and easily sampled. The aims of our study were to examine four distinct populations of *T. thompsonii* using Amplified Fragment Length Polymorphism (AFLP) markers to i) understand general patterns of population genetic diversity and structure, including the relative influence of gene flow, selection and genetic drift, and ii) examine the extent that diversity and structure changed over time. Specifically, this information would help us understand how adequate our efforts have been to conserve *T. thomsonii* using *in situ* and *ex situ* methods and help us determine if *ex situ* resampling intervals should be driven by declining seed viability in storage, or the occurrence of significant change in the genetic structure of *in situ* populations.

## Materials and Methods

### Ethics Statement

Collecting permits for populations growing on US Forest Service land were obtained from the Entiat District Ranger of the Okanogan-Wenatchee National Forest. Verbal permission to collect on state land was obtained from the Natural Areas Ecologist of the Washington Department of Natural Resources. Verbal permission from the landowner (Weaver family) was obtained to collect from private land.

### Species, Study Locations and Sampling

**Figure 1:**
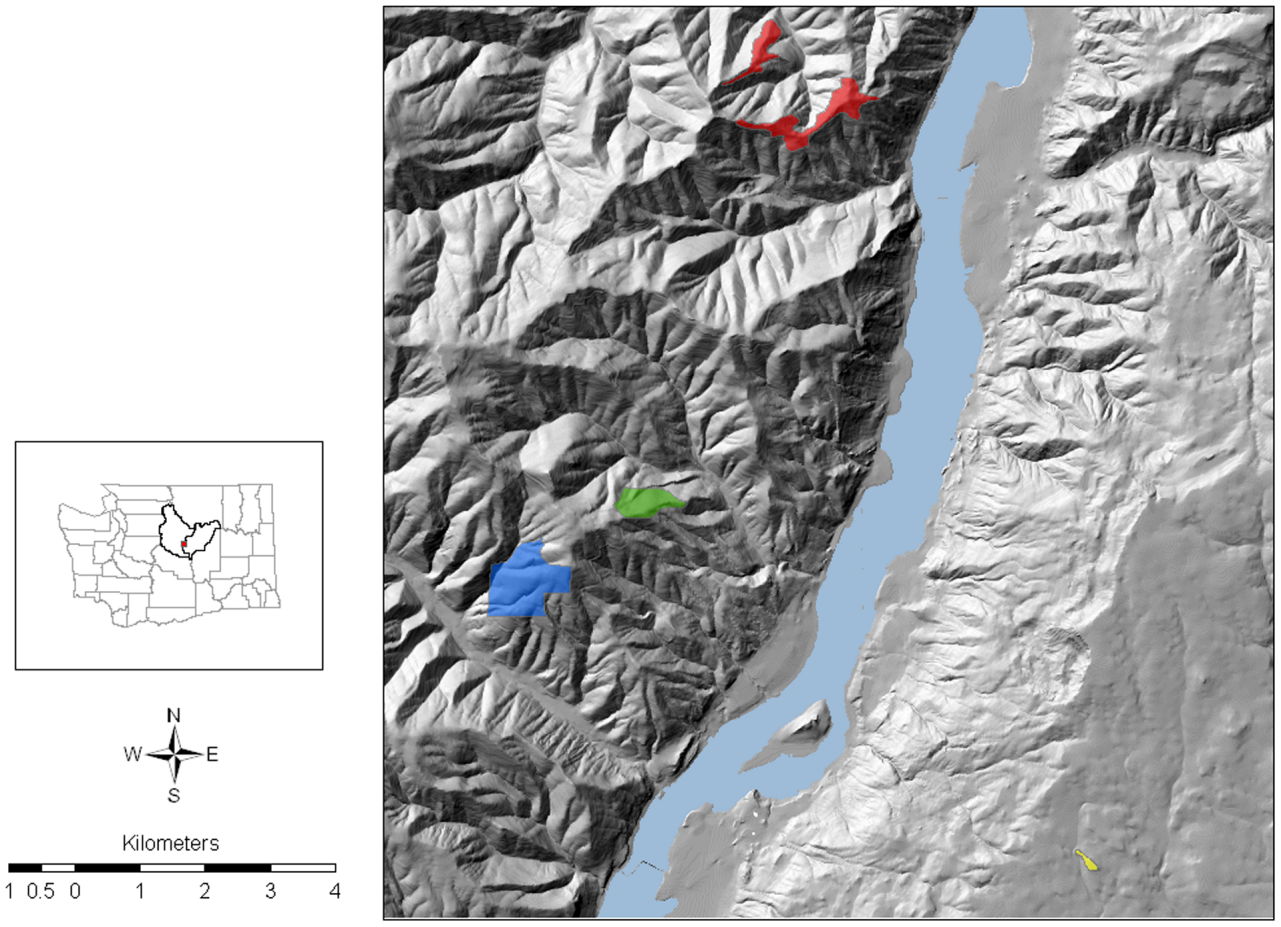
Location of studied populations of *Trifolium thompsonii* in Washington State, USA. Inset map shows Douglas and Chelan county, where Thompson clover is endemic. Four populations were studied: KEYSTONE (red), TENAS (green), RNA (blue) and BADGER (yellow, located east of the Columbia river, and south of RNA).

*T. thompsonii* is reported to occur at 9–14 sites within 187 km^2^ on the east slopes of the Cascade Mountains, in southeastern Chelan and adjacent Douglas County in central Washington State, USA [25]. In 1977, the USDA Forest Service (USFS) established the 81 ha Thompson Clover Research Natural Area (RNA) and in 1993, the Washington Department of Natural Resources (DNR) established the 777 ha Entiat Slope Natural Area Preserve (KEYSTONE). Four sites, including RNA and KEYSTONE were sampled (Fig. 1). Area of each study location was mapped using a GPS (Global Positioning System) in 1995. Study location latitude and longitude were reported in decimal degrees, and elevation, in meters above sea level (masl). Badger Mountain (BADGER) (47.534, −120.2057, 1068 masl) was the only site that lies east of the Columbia River and occurred on private land. RNA (47.566, −120.324, 687 masl) and TENAS (47.581, −120.299, 982 masl) were located due west and northwest (respectively) of BADGER, on the USFS Okanogan-Wenatchee National Forest. KEYSTONE (47.635, −120.278, 1176 masl) was the northern most site. Size of area, habitat classification, and estimated census size of the four populations can be found in Table 1.

**Table 1:** Size and habitat of study locations, population census estimate based on mean plant density, and number of plants analyzed using AFLP markers.

Location	Area (km^2^)	Habitat^a^	Population census		No. plants analyzed		
			<1998	2008	1995	2004	2009
BADGER	0.04	*Artemisia tridentata*/*Agropyron spicatum*	193,368^b^	166,666^d^	18	64	60
RNA	1.01	*Artemisia tridentata-vasayana*/*A. spicatum*	6,075828^c^	1,630,785^e^	19	63	56
TENAS	0.36	*Pseudotsuga menziesii/Calmagrostis rubescens*	7,174,060^c^	5,564,140^f^	0	64	60
KEYSTONE	0.82	*Artemisia tridentata-vasayana*/*A. spicatum*	1,481,738^b^	1,545,595^g^	35	64	48

a Habitat description based on classification of vegetation [27].

b [27].

c [30].

d Washington Natural Heritage Program Report *Trifolium thompsonii* EO 2342-SF 1856.

e Washington Natural Heritage Program Report *Trifolium thompsonii* EO 6503.

f Washington Natural Heritage Program Report *Trifolium thompsonii* EO 3235.

g Washington Natural Heritage Program Report *Trifolium thompsonii* EO 2342.

In 1995 and again in 2004, seed was sampled between July 22 and August 10 from the four sites, and placed in *ex situ* storage (−18°C). In 2009, leaf tissue was sampled from *in situ* populations growing at the four sites. For the purposes of this study, each site was designated as a population, and samples collected from a specific site during a specific year were considered subpopulations. Seed and leaf tissue samples were collected by walking multiple haphazard linear transects of 150 m throughout the study site. Two inflorescence or three young and healthy leaves were sampled from a single plant if it occurred at 5 m intervals along thetransect. This distance was considered sufficient to minimize within family sampling based on observations of seed dispersal [28]. A total of 150 plants were sampled per site. Sampling protocols, which also included bulking seed from maternal lines, were in accordance to standard collecting protocols for efficient *ex situ* conservation of plant genetic resources. Seed was cleaned and stored in a −18°C freezer. For the analysis, seed was scarified and subjected to an 18 hour water rinse that we have found effective in overcoming seed dormancy. Seeds were germinated in petri dishes and fresh and healthy seedlings were transported to the molecular laboratory where seedling tissue was frozen at −80°C and lyophilized in a Vertis Lyo-Centre Freeze Dryer (SP Industries, Gardiner NY). A third of the 1995 seedlings were inadvertently frozen during transit and had to be discarded. The 2009 leaf samples were placed in envelops, kept at 4°C for 24 hours then placed in a −80°C freezer. Leaf tissue was lyophilized using the same procedure as the seedling tissue.

### DNA Isolation and AFLP marker generation

Although less informative than codominant markers, AFLP markers have been used widely to investigate population genetic structure in insect-vectored out crossing species (i.e. [31]–[34]). As with any molecular marker system, care needs to be taken to minimize genotyping errors so methods used to isolate DNA and generate AFLPs incorporated rigorous protocols, internal standards and replication to ensure genotypes determined by the marker analysis correspond to the real genotypes being examined [35].

Freeze-dried seedlings or leaf tissues were pulverized in a SPEX SamplePrep 2000 Geno/Grinder, and DNA extraction was automated using the Wizard Magnetic 96 DNA Plant System (Promega). The AFLP analysis was based on individual plants germinated from seed collected at the same locations in 1995 and 2004, and on leaf samples of individual plants collected from the same locations in 2009.

AFLP markers were generated using locally developed procedures based on technology by [36]. Double restriction digestion was done in a 25 ul reaction containing 250 ng of DNA, 1X NE Buffer 4, 1X Purified BSA and 5.0 U each of EcoRI and MseI restriction enzymes (New England BioLabs). Fifteen ul of the restriction digest reaction was run on a 1.5% agarose gel to verify the completion of digestion.

Adapter sequences (EcoRI-Fwd, 5′- ctc gta gac tgc gta cc; EcoRI-Rev, 5′- aat tgg tac gca gtc tac; MseI - Fwd, 5′- gac gat gag tcc tga g, and MseI-Rev, 5′- tac tca gga ctc at) were obtained through Eurofins MWG/Operon (Huntsville, Alabama). Each adapter pair was adjusted to 100 pM/ul (EcoRI) and 200 pM/ul (MseI), mixed in equal amounts, and allowed to anneal for 1 hour at 37°C and cooled to room temperature. The annealed pairs were then diluted to 5 pM/ul (EcoRI) and 50 pM/ul (MseI), aliquoted to 100 ul amounts and frozen for future use.

Ligation was done at 20°C for 2 hours in a 20 ul reaction containing 10 ul of the remaining restriction digest, 1X T4 Ligase Buffer (New England BioLabs), 5 pMoles EcoRI adapter, 50 pMoles MseI adapter, 0.5 mM ATP, and 80 cohesive end Units of T4-ligase (New England Biolabs). The completed ligation reaction was diluted 10∶1 and used for the Pre-amplification. Pre-amplification and selective amplification were done on an ABI 9700 thermocycler using cycling programs prescribed by [36] but in 10 ul reactions. The pre-amplification product was diluted 10∶1 and 2 ul used for selective amplification. Four separate primer pairs were used for selective amplification (Eacg/Mctg, Eaca/Mctc, EacaMcag, and EaggMctg, where the last 3 letters indicate the selective nucleotides following the EcoRI and MseI primer sequences) since they provided clear reproducible bands and were sufficiently polymorphic to show variation within and between populations. Marker fragments were separated on a LI-LOR 4300 DNA Analyzer (LI-COR Biosciences). Unambiguous bands were identified and scored as either present or absent. To estimate genotyping error, four replicates were run on eight individual plants each, from two contrasting sites. Genotyping error was estimated at less than 1%.

### Data Analysis

Since we had unequal sample sizes (Table 1.), a subsampling approach was used to determine if uneven sample sizes would bias our analysis [37]–[38]. Sub sampling was performed in R (script available upon request) by modifying the Diversity function in AFLPdat [39] to sub sample a random sample of 18 individuals (the minimal sample size), from each subpopulation and calculate Nei’s gene diversity and proportion of polymorphic markers. This was reiterated 100 times and means and standard deviation were calculated. Since observed and sub sampled data were consistently the same (Table S1.), and suggested that bias due to unequal sample size was low, the subsequent analysis was based on the observed data.

For each year, band patterns were calculated for overall population and subpopulations for the following: total number of bands (NB), number of common bands (CB) (frequency >0.05), number of rare bands (RB), (frequency <0.05), number of not widely shared bands (NWS), (common bands found in 25% or fewer populations) and number of private bands (PB). Allele frequencies were estimated using AFLPsurv 1.0 [40] based on a Bayesian method with non-uniform prior distribution [41]. Hardy-Weinberg equilibrium was assumed since Thompson clover is a highly out crossing wild species. Allele frequencies were used to estimate percent polymorphic loci at the 5% level and Nei’s gene diversity and its standard error was estimated for each subpopulation. T tests were carried out to determine if genetic diversity was significantly different among the four populations for each sampled year (i.e BADGER04, RNA04, TENAS04, KEYSTONE04), and across the three sampled years for each population (i.e. BADGER95, BADGER04, BADGER09). A Bonferroni correction was made to ensure an overall critical p-value of 5%. To examine genetic differentiation, AFLPsurv 1.0 was used to estimate overall population Fst at the four locations. It was also used to test the null hypothesis that there was no genetic differentiation among the four populations for each sampled year, and across the three sampled years for each population. Five thousand permutations were used.

To further examine population differentiation and partitioning of genetic variance, a euclidean distance metric was estimated and used for an analysis of molecular variance (AMOVA) to calculate Phi statistics (which is analogous to Fst), using GENALEX 6.5 ([42]–[43]. Pairwise PhiPT was estimated among the four populations by combining the 2004 and 2009 data. Probability values were based on 999 permutations. The AMOVA was also used to examine the distribution of variation among location (PhiRT) (i.e. BADGER, RNA, KEYSTONE), and among subpopulations (i.e. 1995, 2004, 2009) within location (PhiPR). Since TENAS lacked data for 1995, it was not included in this analysis. Significance of the AMOVA was tested using 999 permutations.

To examine genetic distance among the spatially and temporally sampled populations, Nei’s D_A*in*_ [44] which does not involve an evolutionary model, was estimated using PowerMarker 3.25 [45]. A cluster analysis was conducted on all subpopulations using the neighbor joining algorithm to construct a tree from the distance matrix. The program was also used to calculate 1000 bootstrap distance matrices which were imported into the CONSENSE program of PHYLIP [46] to build a majority rule consensus tree.

Population structure was also examined using the software STRUCTURE v2.3.3 [47]–[50]. Ten replications with a burn-in of 20,000 iterations followed by 20,000 additional iterations were used at each K level until results indicated lowered and erratic values for P(X|K). The parameter set included the ADMIXTURE model with allele frequencies correlated, RECESSIVE ALLELES model, USEPOPINFO and STARTATPOPINFO turned on with 11 subpopulations labeled (each location/year), and the LOCPRIOR model. Average Q-plots over the ten replications were calculated using the ancillary software CLUMPP [51], and graphic displays of population structure were developed from the q-frequencies of the mean of ten runs using DISTRUCT software [51].

To examine gene flow on a current timescale we carried out two different assignment tests. The first used AFLPOP 2.0 [52] which uses a modified allocation method [53] to compute the log likelihood of an unknown individual’s allelic phenotype in each subpopulation based upon the frequency of the dominant band (presence) at each locus. Each unknown individual is then allocated into a source subpopulation with the greatest log likelihood. The minimal log-likelihood difference for the allocation of an individual to a population was set at 0.5. This is a conservative threshold in that assignment of an individual to a subpopulation was not made unless the probability of the given assignment was 5 times more likely than the next most probable assignment. If this threshold was not met, the individual was not assigned to any subpopulation and were designated as ‘criteria not met’ (CNM). The CNM category does not imply that an individual does not belong to a subpopulation, but that there are two or more subpopulations with similar probabilities of assignment, and hence minimal log-likelihood difference is less than the designated threshold. We also used STRUCTURE v2.3.3 to identify potential migrants between populations taking into account the source population of the sample. We set GENSBACK = 2, which tests for evidence of ancestry for two generations.

To examine the presence of historic gene flow, we tested for isolation by distance among the 4 populations for each year separately using GENALEX 6.5 [43]. A geographic distance matrix was computed based on latitude and longitude coordinates and correlations with the genetic distance matrix were tested using a Mantel test with 9999 permutations [54].

Although genetic differentiation can result from limited gene flow, forces of selection can play an important role in determining the rate of population differentiation. Selection can be detected by identifying loci that have F_st_ values that fall outside the F_st_ range for neutral loci. We used the outlier method of BayeScan 2.1 [55], that uses a Bayesian method to infer the posterior probability of each locus being under selection by defining and comparing two alternative models; including and excluding the effects of selection [56]–[57]. We used 20 pilot runs of 2000 iterations followed by a burn-in of 50 000 iterations and 100,000 iterations, and used a thinning interval of 10. Loci were considered under selection if the posterior odds (PO) was greater than 10 [56]. Subpopulations were examined since the main assumption of the underlying model is that populations exchanging migrants contribute to a common pool of migrants [56]. This assumption would have been violated if we combined data across years. We also dropped out loci whose overall frequency was <0.05, as recommended by the manual. We also used DFDIST as a second outlier detection method, implemented in MCHEZA [58]. MCHEZA allows for the use of dominant markers and models neutral distribution through coalescent simulations under a symmetric island model parameterized by the observed data of neutral loci (i.e. non-neutral loci are dropped out). The simulated neutral distribution is then used to identify individual loci that fall outside the neutral range. For each program we conducted three independent runs and corrected for multiple testing by setting a false discovery rate (FDR) <0.1.

The occurrence of genetic drift can also influence genetic diversity, the rate unfavorable alleles are fixed, and the efficiency of selection [3]. Variance effective population size (N*e*_V_) is an important parameter used to assess genetic drift [59]. Since we had temporally spaced samples, we estimated contemporary N*e*_V_ using a method developed for dominant markers by [60]. Fixed alleles, alleles identified as under selection, and those with a frequency ≤0.2 were eliminated prior to estimation of N*e*_V_ [60]. Because Thompson clover requires several years of establishment prior to seed set, the samples were estimated to be 3 (1995–2004) and 2 (2004–2009) breeding generations apart. Since sample sizes were small and *N_e_* reasonably large, 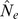 was estimated using 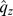 in all cases.

## Results

### Patterns of genetic diversity and differentiation

The four primer pairs revealed a total of a 129 readily discernible polymorphic bands. Table 2 summarizes the AFLP alleles from the *ex situ* samples collected in 1995 and 2004 and the *in situ* sample from the same populations in 2009. Across all populations, the number of common alleles was the same in all three sampling periods; however, more rare alleles were sampled in 2004 and 2009, compared to 1995. In the individual populations, the proportion of rare alleles was greater in TENAS and KEYSTONE, compared to BADGER and RNA, and this pattern was observed in all three sample years. The occurrence of private alleles was very low; however, there were the occurrence of common bands that were not widely shared among populations. KEYSTONE had the lowest proportion of polymorphic loci, followed by TENAS. BADGER and RNA had the highest percentage of polymorphic loci and were comparable. For all populations, percent polymorphic loci did not change over the fifteen years, with the exception of BADGER, where 2004 (79.8) was greater than 1995 (75.2) and 2009 (76.0).

**Table 2:** AFLP band patterns and % polymorphic loci for four populations and for the combined populations (POP), sampled for three time periods.

BAND	BADGER	RNA	TENAS	KEYSTONE	POP
**1995**					
NB	110	109	–	109	121
CB	110	109	–	102	109
RB	0	0		7	12
NWS	2	0	–	0	0
PB	1	0	–	0	1
%P	75.2	76.7	–	69.0	73.6
**2004**					
NB	111	118	112	114	126
CB	105	109	99	100	109
RB	6	9	13	14	17
NWS	2	0	3	1	0
PB	0	0	0	0	0
%P	79.8	77.5	72.9	69.0	74.8
**2009**					
NB	109	118	113	109	127
CB	107	112	102	97	109
RB	2	6	11	12	18
NWS	3	3	2	1	0
PB	0	0	0	0	1
%P	76.0	77.5	72.1	67.4	73.2

NB = total number of bands; CB = Common bands (number of different bands with a frequency >0.05); RB = Rare bands (number of different bands with a frequency <0.05); NWS = Not widely shared bands, (common bands found in 25% or fewer populations); PB = number of private bands; %P = percent polymorphic loci at the 5% level, expressed as a percentage.

Across all populations there was no significant change in gene diversity (P<0.05) from 1995 to 2009. Table 3 summarizes the gene diversity statistics for each subpopulation. In 1995, KEYSTONE had significantly less diversity than BADGER and RNA. TENAS and KEYSTONE also had significantly less diversity in 2004 and 2009 compared to BADGER and RNA. Interestingly, the BADGER 1995 subpopulation, based on eighteen individual plants, had significantly greater gene diversity than 2004 and 2009 subpopulations based on >50 individual plants. Diversity measurements were the same for RNA, TENAS and KEYSTONE regardless of the year sampled. The four populations showed significant but low genetic differentiation (P<0.001). Populations located close together had less differentiation than populations located further apart. The overall estimate for F_st_ was 0.05. Pairwise F_st_ between geographically close sites ranged from.03 in 1995 and 2004 to.04 in 2009. In contrast, F_st_ between BADGER and KEYSTONE, which were the furthest away, ranged from.07 in 1995, .05 in 2004 and.06 in 2009. Population pairwise PhiPT values showed a similar result (Table 4).

**Table 3:** Expected heterozygosity (H_e_) under Hardy-Weinberg genotypic proportions for *ex situ* samples collected in 1995, recollected in 2004, and *in situ* populations sampled in 2009.

Location	1995	2004	2009
BADGER	0.292 aa	0.276 ab	0.275 ab
RNA	0.278 aa	0.271 aa	0.274 aa
TENAS	–	0.247 ba	0.250 ba
KEYSTONE	0.250 ba	0.250 ba	0.242 ba

Differences in first letter correspond to significant differences in H_e_ among the four locations within the same year. Differences in second letter correspond to significant differences in H_e_ within location across the three years sampled (Bonferroni correction for testing seven hypotheses based on overall critical p-value of 5% is ≤0.007).

**Table 4:** Geographic distance (km) between each location (above diagonal) and population pairwise PhiPT values (below diagonal) based on samples collected in 2004 and 2009 (Values significant at P<0.001).

Population	BADGER	RNA	TENAS	KEYSTONE
BADGER	–	10.07	9.09	13.21
RNA	0.068	–	2.47	8.21
TENAS	0.081	0.046	–	6.31
KEYSTONE	0.099	0.087	0.085	–

### Spatial and temporal structure

**Figure 2:**
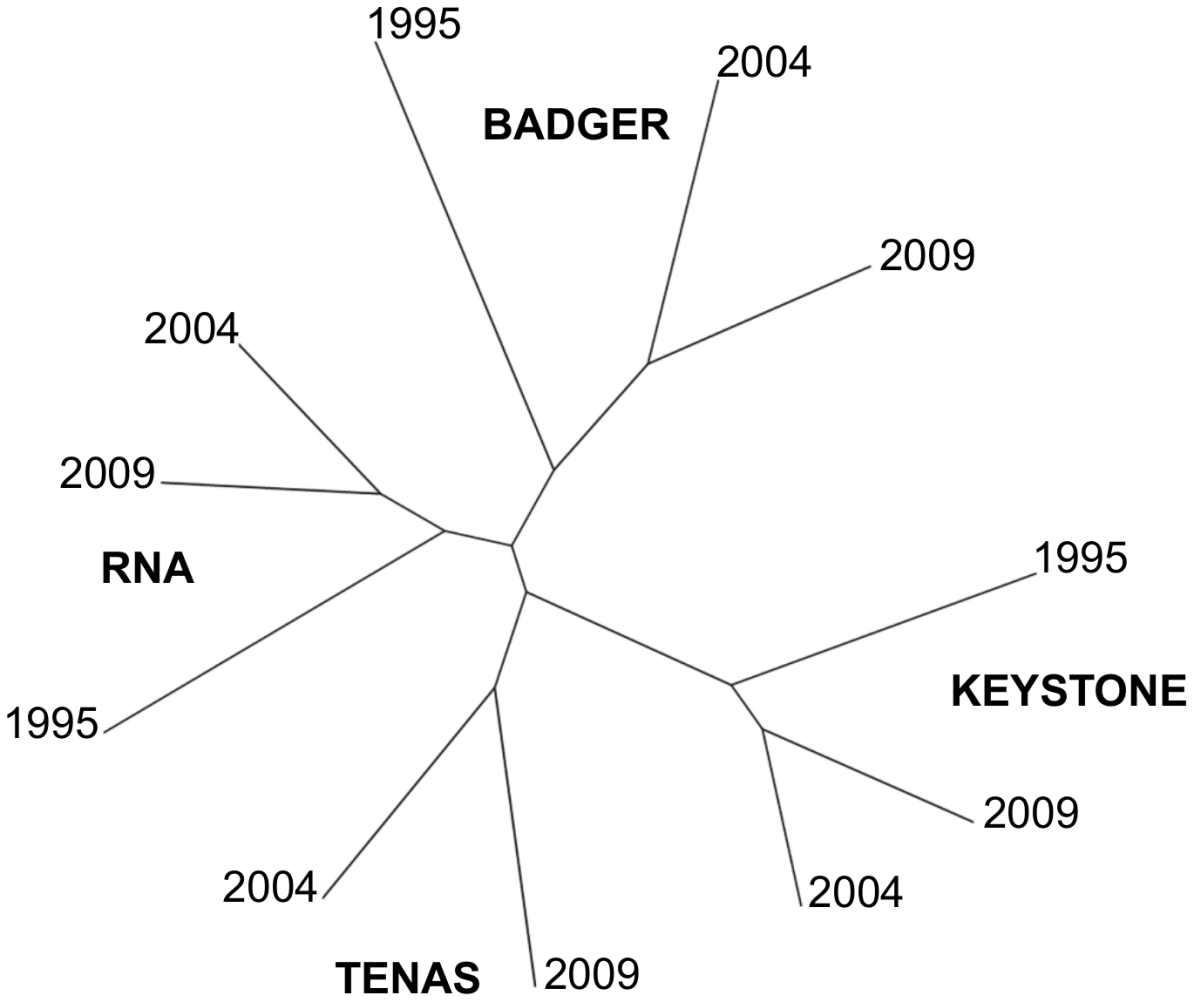
Neighbor-joining tree showing genetic relationship among Thompson clover. Relatedness of four populations of Thompson clover sampled in 1995, 2004, and 2009, based on Nei’s genetic distance estimated using AFLPs.

Based on the AMOVA analysis, 90% of the variation occurred within populations. Seven percent of the variation occurred among populations and 3% occurred among subpopulations collected within each location (P-value <0.01 with 999 permutations) (Table 5). The neighbor-joining tree based on Nei’s genetic distance is shown in Fig. 2. Similar to the AMOVA, the tree suggested that spatial structure was more significant than temporal structure. Subpopulations collected from the same location clustered together; however, populations sampled in 1995 were less similar to populations sampled in 2004 and 2009, which were more closely related. Genetic structure associated with geographic distance was also evident. Subpopulations collected at KEYSTONE, the northern-most site, were genetically more distant compared to the subpopulations collected at TENAS, RNA, and BADGER. However, KEYSTONE subpopulations were more closely related to TENAS, which is closer, geographically, and least related to BADGER, which is the site furthest away.

**Table 5:** Nested analysis of molecular variance (AMOVA) based on 129 polymorphic loci based on populations sampled at three locations (BADGER, RNA, and KEYSTONE), for three different years (1995, 2004, 2009).

AMOVA	df	MS	Variation	% variation	Phi	P-value
Location	2	225.538	1.229	7	0.069	0.01
Year/location	6	40.166	0.529	3	0.032	0.01
Subpopulation	431	16.085	16.085	90	0.099	0.01
Total	439		17.844	100		

**Figure 3:**
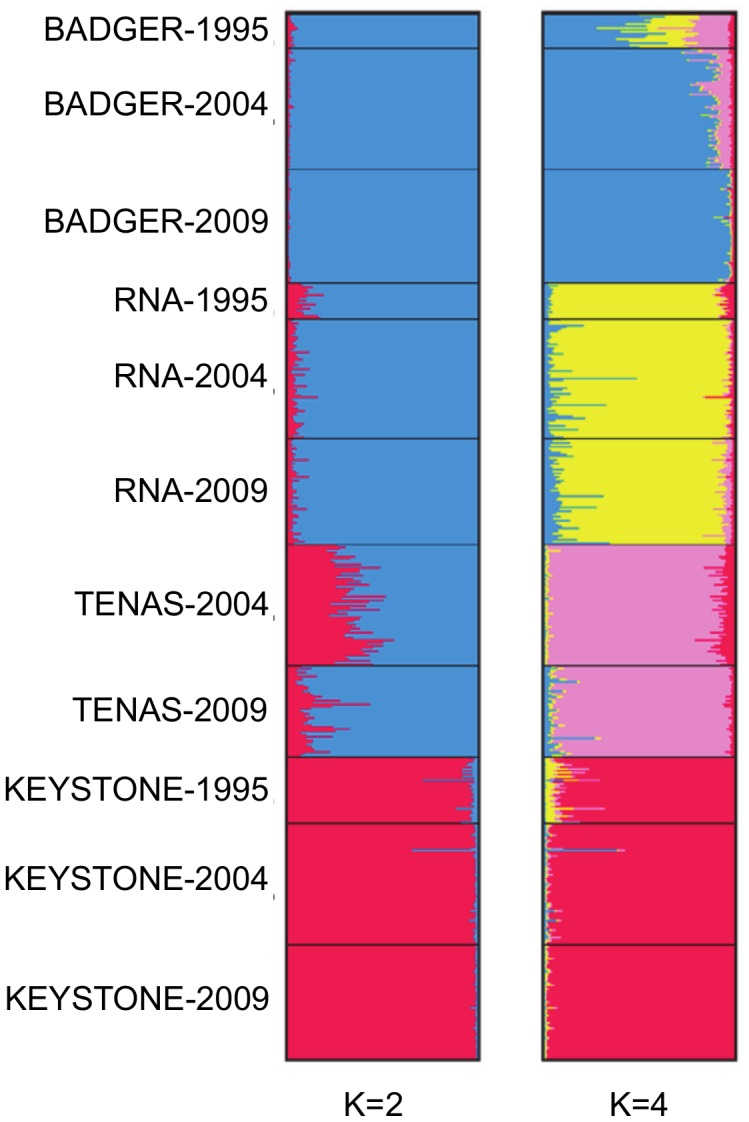
Q-plots based on AFLP analyses. Bayesian analysis using the software STRUCTURE suggested most likely groups of K = 2 and K = 4.

The STRUCTURE analysis also supported strong spatial structure. The plot of P(X|K) indicated that K = 2 and K = 4 were the most likely groupings (Figure S1). Fig. 3 shows Q-plots for each K group which are useful for visualizing population genetic structure and the presence of admixture. At K = 2, the KEYSTONE population stood out as distinct from BADGER, RNA and TENAS, in all three years. As with the cluster analysis, geographic structure was apparent. Looking at the level of admixture, the proportion of alleles belonging to the KEYSTONE group decreased from TENAS to BADGER, reflecting increasing geographic distance. At K = 4, geographic differentiation between the four populations was apparent. Although not as distinct, variation across sampling year was apparent. For example in BADGER 1995, admixture with RNA and TENAS was apparent, but the proportion of alleles belonging to RNA was much less in BADGER 2004 and BADGER 2009. The proportion of alleles belonging to TENAS was somewhat less in BADGER 2004, and much less in BADGER 2009.

### Evidence for gene flow

The assignment tests suggested that limited gene flow did occur between the four populations. With AFLPOP, (Table 6), individuals were assigned to the correct source population at a rate that ranged from 80 to 32%. Generally individuals from subpopulations collected at the same location were assigned with a similar level of success to the correct population. TENAS had the highest success, averaging 71% across 2004 and 2009. RNA had the lowest success rate, averaging 43% across the three sampling years. Seventy six percent of assignment events between locations were between locations that were adjacent. However, there was evidence for long distance gene flow from BADGER to RNA, RNA to KEYSTONE, BADGER to TENAS and TENAS to BADGER. Migration between populations did not appear symmetrical. There were twice as many genotypes from RNA assigned to BADGER, then from BADGER to RNA. There were twice as many genotypes from TENAS assigned to RNA, as there were from RNA to TENAS. Only one genotype from KEYSTONE was assigned to a location other than KEYSTONE. Mantel tests for isolation by distance found a significant and positive relationship between genetic distance and geographic distance (P<0.001, 9999 permutations) which suggested that gene flow between the four populations was limited, but did occur based on the evidence provided by the assignment tests.

**Table 6:** Assignment of 551 individuals from seven subpopulations (columns) to source populations (rows) to detect gene flow between four locations over 15 years.

Allocated to	BAD95	BAD04	BAD09	RNA95	RNA04	RNA09	TENAS04	TENAS09	KEY95	KEY04	KEY09	Total
BAD 1995	11	0	0	0	0	0	0	0	0	0	0	11
BAD 2004	0	40	7	0	1	2	0	0	0	0	0	50
BAD 2009	0	8	33	0	2	1	0	1	0	0	0	45
RNA 1995	1	0	0	8	1	0	0	0	0	0	0	10
RNA 2004	0	1	0	3	31	9	2	0	0	0	0	46
RNA 2009	0	0	1	0	8	22	0	4	0	0	0	35
TEN 2004	1	1	0	0	0	0	51	1	1	0	0	55
TEN 2009	0	0	0	0	1	2	6	30	0	0	0	39
KEY 1995	0	0	0	0	0	0	1	1	19	4	0	25
KEY 2004	0	0	0	0	0	0	0	0	5	21	3	29
KEY 2009	0	0	0	0	1	0	0	0	0	14	35	50
CNM^a^	5	14	19	8	18	20	3	11	10	25	22	155
# IND	18	64	60	19	63	56	63	48	35	64	60	551

a Criteria not met (CNM) to meet minimal log-likelihood difference of 0.5 for the allocation of an individual to a population.

### Extent of selection and genetic drift

Outlier loci detection suggested that most of the markers we examined were neutral but there was some signature of selection. BayeScan 2.1 identified no significant outliers among the 4 K groups at either prior odds of 10∶1 or 1∶1 for all three years sampled. However, DFDIST showed a high probability (>95%) for divergent selection for 9, 7 and 9 loci in 1995, 2004 and 2009, respectively. In one instance, the same locus experienced selection in all three years, while a second locus experienced selection in 1995 and 2004, and a third, experienced selection in 2004 and 2009.

When we estimated effective population size, we found that NeV declined in RNA from 163 to 113. However, it increased in BADGER and KEYSTONE (Table 7). This coincided with the census population numbers (Table 1), that suggested a drop from 6 million plants in 1998 to slightly more than 1.5 million plants in 2008 at RNA while BADGER and KEYSTONE remained relatively stable.

**Table 7:** Estimated population sizes (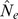) of the four locations over the three sampling years.

Years	BADGER	RNA	TENAS	KEYSTONE
Loci	66	77	83	77
1995–2004	30	163	–	105
2004–2009	44	113	57	156

## Discussion

### General patterns of genetic diversity and population structure

Although *T*. *thompsonii* is a narrowly distributed endemic species, our results showed that it did not have a narrow genetic base. The overall level of genetic diversity (0.26) was similar to comparable AFLP studies on widespread perennial insect-pollinated congeners, T. *montanum* L., (0.23), [61], and *T*. *alpinum* L., (0.24), [62], and higher than *T*. *repens*, (0.1) [63]. Average percent polymorphic loci was higher (73%) than reports for *T. montanum* (58%) [61] and *T. repens* (31%) [63]. Diversity estimates were also similar to comparable AFLP studies in non related herbaceous perennial insect pollinated species, such as *Silene chlorantha* (Willd.) (0.20) [34], *Abronia alpina* Brandegee (.28) [64], and *Echinacea laevigata* (Boynton and Beadle) Blake (0.26) [65].

Among the four populations, KEYSTONE and TENAS, the northern most populations, had less overall diversity and a lower percent of polymorphic loci compared to BADGER and RNA. They also had about twice as many rare bands. An explanation may be that these populations are relicts of the leading north edge of the species during the Pleistocene glaciations. These populations lie due south of the furthest extent of the Okanogan lobe of the Cordillera ice sheet, and although major valley glaciers extended eastward from the Cascade Range, the closest stopped at the upper Entiat river, approximately 10 km west of KEYSTONE [28], [66]. Simulation studies have shown that neutral genetic variation can be lower along the leading edge of a species range due to founder effects and allele surfing [67], [68].

BADGER was the only population that showed a significant decline in genetic diversity from 1995 to 2004. It was also the only population where a private band was detected. Considering that this population is located in open shrub steppe habitat, as opposed to the other three populations that are located in the forest transition zone, it may contain unique alleles that help it persist in a drier, hotter environment. Although resampling suggested that uneven sampling sizes did not bias our genetic estimates, we would expect if bias was present, sampling too few plants would have underestimated diversity. Although we sampled fewer plants in 1995, compared to 2004 and 2009, 20 individuals were determined to be adequate to assess genetic variation in *T. repens*, also an insect-pollinated outcrossing perennial species [69]. Our results were also consistent with field observations. Although the census population size of BADGER has remained relatively stable, we observed that the population was protected from grazing in 1995, but was not protected when we returned in 2004 and 2009. Because BADGER is in a unique habitat, compared to other populations, and does not have State or Federal protection, close monitoring is warranted to ensure this population does not continue to decline in the future.

### Relative influence of gene flow, selection and genetic drift

Although genetic differentiation between the four populations was significant (albeit weak), as was our test of isolation by distance, there was sufficient evidence to suggest that gene flow did occur among the studied populations.

The assignment test suggested infrequent gene flow occurred, despite the distance between populations. The asymmetrical flow of genes was concordant with site specific characteristics, in terms of climate and topography. The occurrence of twice as many migration events from RNA to BADGER, compared to BADGER to RNA supported the hypothesis that prevailing northwesterly winds may disperse fruits of *T*. *thompsonii* or contribute to the long distance movement of insect pollinators, since RNA is located northwest of BADGER [28]. Gene flow also occurred more frequently from TENAS to RNA than from RNA to TENAS. Although separated by 2.5 km, TENAS and RNA occur along the same road. However, TENAS is at a higher elevation, so seed and pollen would more likely move downhill, from TENAS to RNA. Results from the assignment tests reflected the influence of distance as well. Gene flow appeared to have occurred between KEYSTONE and TENAS, separated by 6.31 km, but was restricted between KEYSTONE and RNA, (8.21 km), and did not occur between KEYSTONE and BADGER (13.21 km). The usefulness of topographic data to infer gene flow in wild *Trifolium* populations has been reported and has been concordant with data generated from morphologic [70], isozyme [71], RAPD [70] and AFLP [63] markers. This supports the use of maps and satellite images to infer gene flow due to the occurrence of landscape barriers or corridors that influence the connectivity between populations.

Although there was some evidence that selective forces were responsible for influencing Fst values for some of the markers we examined, it was only for a few. However one marker was identified as being under divergent selection in all three years examined, and may be associated with an adaptive trait. Although succession has been occurring in the area where RNA, TENAS and KEYSTONE are located since the 1988 fire, our marker set did not detect any overall temporal trends in changing selection pressure. Studies similar to [72] would be useful to examine this in further detail since it has direct relevance to recollecting intervals.

There was limited evidence for genetic drift although we did detect a decline in effective population size in RNA, which was concordant with consensus data. However, we did not detect a decrease in genetic diversity. Our study suggested that further monitoring is warranted in RNA to ensure population decline does not have a negative impact since the population is the largest and oldest *in situ* reserve of Thompson clover.

Altogether, we tracked the genetic structure of 4 different populations of Thompson clover over 15 years. With the exception of BADGER, the populations did not appear to have undergone substantial change during the time period of our study. Changes in genetic structure of the RNA, TENAS and KEYSTONE populations due to succession following the 1988 fire, were not evident. Although gene flow was evident, admixture tended to decline over time, especially in BADGER and KEYSTONE. This suggested that despite the occasional influx of migrants, individual populations tended to return to equilibrium, as unadapted genotypes were selected against. Overall, spatial differences among the 4 study locations accounted for more than twice the variation as temporal differences within study locations. These results suggested that recollecting intervals for *ex situ* samples of Thompson clover could be greater than 15–20 years.

## Conclusion

In this paper, we report the spatial and temporal genetic variation and population substructure revealed by AFLP markers in T. *thompsonii* populations sampled from four diverse locations over a 15 year time period. Although there was some evidence for gene flow, selection and drift, there was no difference in overall diversity between *ex situ* samples collected in 1995 and 2004 and *in situ* samples collected in 2009. Only one population showed a decline in genetic diversity over the 15 years studied. Our results echo other reports that have found that perennial out crossing species generally showed less difference in genetic diversity between *in situ* and *ex situ* accessions compared to annual, self-pollinated species [73].

We can use the lessons we learned in our study to formulate some general guidelines to help ensure *ex situ* samples of insect-pollinated herbaceous perennial species provide clear snap shots of *in situ* populations. Our study suggested that a successful *ex situ* collecting strategy is to collect from a large number of plants (>150 plants), equalize maternal contribution by sampling the same number of heads per plant, and sample along random transects with sufficient space between plants to minimize intrafamilial sampling. We also learned that long distance dispersal can occur, and appeared to play an important role in limiting the extent that our four populations diverged from one another. Long distance gene flow by insect pollinators not only maintained genetic connectivity, but can also help in maintaining and increasing effective population size [74], [75]. Only one immigrant every other generation is needed to prevent differentiation due to genetic drift [76], [77]. Long distance dispersal of pollen by insects can be highly variable since it is dependent upon plant species, pollination vector and the environment [74], so the effective conservation of insect pollinated species requires conserving not only the plant species, but pollinator diversity and abundance [78]. Our results suggested that as a general guideline, for a given geographic area, the benefits of long distance gene flow may allow us to conserve the intraspecific diversity of insect pollinated species (especially populations that occur in similar habitats), with fewer populations, since populations are likely to be weakly divergent. In contrast, self pollinated species, especially annuals, may need more populations protected, since we would expect individual populations to be strongly divergent, due to isolation by distance.

The most important lesson we can apply to the effective *ex situ* and *in situ* conservation of PGR is the value of comparing the genetic structure of *ex situ* and *in situ* samples. Quantifying genetic change over time not only allows genetic resource managers to validate conservation protocols, develop appropriate recollection intervals, but provides them with an early detection mechanism for identifying problematic conditions that can be addressed to prevent the local extinction of vulnerable *in situ* populations.

## Supporting Information

Figure S1 The plot of P(X|K) indicated that K = 2 and K = 4 were the most likely groupings.
(TIF)

Table S1 Results from resampling to determine unequal sample bias.
(DOCX)
