# Supplementary figures and images for: Conserving Plants in Gene Banks and Nature: Investigating Complementarity with Trifolium thompsonii Morton
Source: PLoS One. 2014 Aug 14;9(8):e105145. doi: 10.1371/journal.pone.0105145 (PMC4133347; doi:10.1371/journal.pone.0105145)

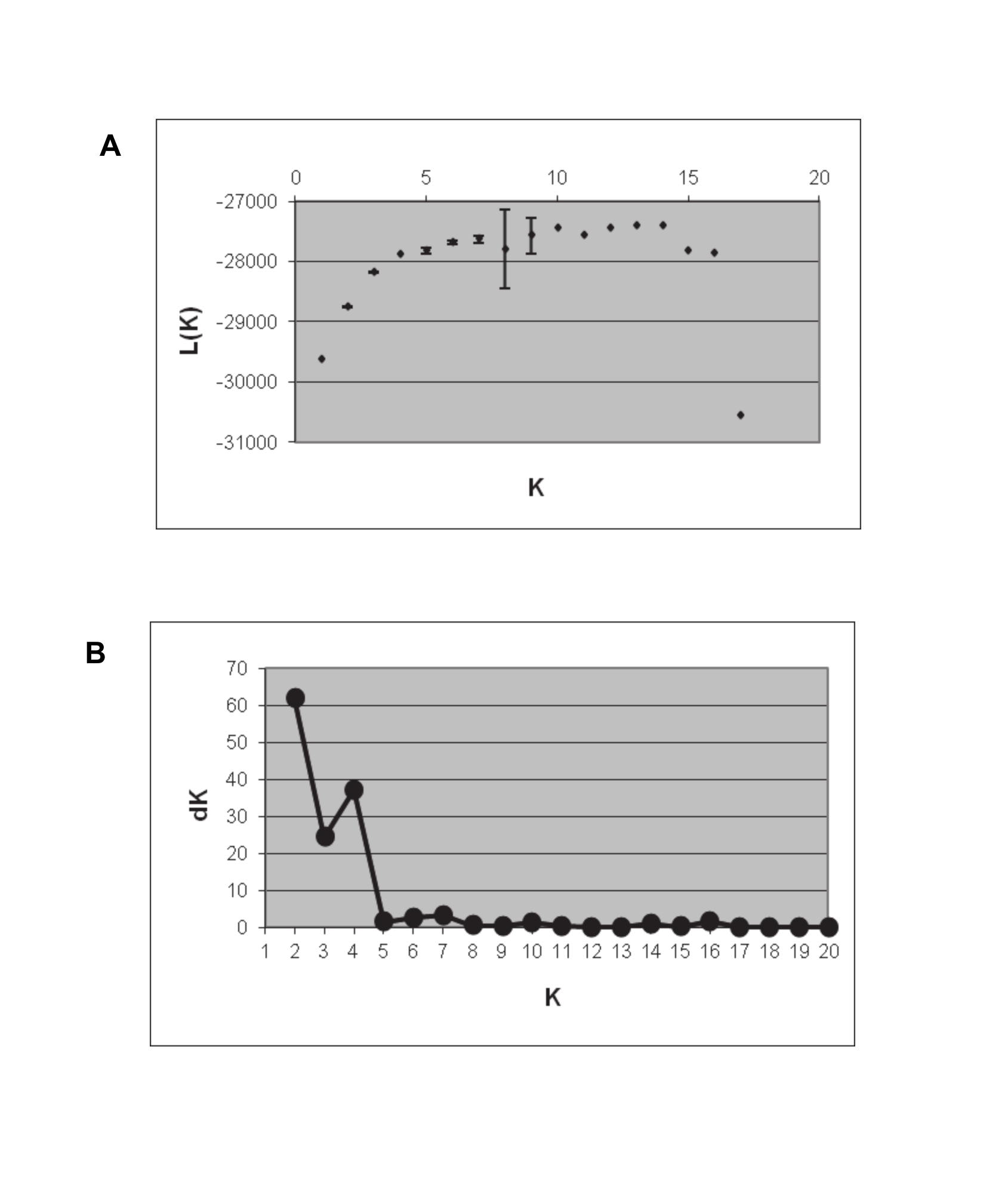

Supplement: Figure S1 — The plot of P(X|K) indicated that K = 2 and K = 4 were the most likely groupings. (TIF) [file pone.0105145.s001.tif]
