# Supplementary material for: Conserving Plants in Gene Banks and Nature: Investigating Complementarity with Trifolium thompsonii Morton
Source: PLoS One. 2014 Aug 14;9(8):e105145. doi: 10.1371/journal.pone.0105145 (PMC4133347; doi:10.1371/journal.pone.0105145)
Supplement: Table S1 — Results from resampling to determine unequal sample bias. (DOCX) [file pone.0105145.s002.docx]

Table S1. Results from resampling to determine unequal sample bias

| **Population** | **PopSize** | **Original GD** | **SampleSize** | **Resample No.** | **ResampleMean GD** | **STdev** | **Max** | **Min** |
| --- | --- | --- | --- | --- | --- | --- | --- | --- |
| Badger1995 | 18 | 0.2643 | NA | NA | NA | NA | NA | NA |
| Badger2004 | 64 | 0.2572 | 18 | 100 | 0.257 | 0.006 | 0.273 | 0.239 |
| Badger2009 | 60 | 0.2523 | 18 | 100 | 0.253 | 0.008 | 0.273 | 0.237 |
| Keystone1995 | 35 | 0.2258 | 18 | 100 | 0.225 | 0.007 | 0.245 | 0.206 |
| Keystone2004 | 64 | 0.2254 | 18 | 100 | 0.225 | 0.008 | 0.242 | 0.209 |
| Keystone2009 | 60 | 0.2246 | 18 | 100 | 0.225 | 0.007 | 0.241 | 0.210 |
| RNA1995 | 19 | 0.2654 | 18 | 100 | 0.265 | 0.002 | 0.269 | 0.262 |
| RNA2004 | 63 | 0.2692 | 18 | 100 | 0.268 | 0.007 | 0.285 | 0.253 |
| RNA2009 | 57 | 0.2788 | 18 | 100 | 0.270 | 0.008 | 0.285 | 0.243 |
| Tenas2004 | 64 | 0.2293 | 18 | 100 | 0.229 | 0.008 | 0.246 | 0.213 |
| Tenas2009 | 48 | 0.2351 | 18 | 100 | 0.235 | 0.006 | 0.249 | 0.222 |

GD- Nei’s Genetic Diversity
